# Clustering of Social Determinants of Health as an Indicator of Meaningful Subgroups within an African American Population: Application of Latent Class Analysis

**DOI:** 10.3390/ijerph21060676

**Published:** 2024-05-24

**Authors:** Sumihiro Suzuki, Joshua Longcoy, Zeynep Isgor, Elizabeth Avery, Tricia J. Johnson, Eric Yang, Elizabeth B. Lynch

**Affiliations:** 1 Department of Family and Preventive Medicine, Rush University Medical Center, Chicago, IL 60612, USA; elizabeth_avery@rush.edu (E.A.); elizabeth_lynch@rush.edu (E.B.L.); 2 Center for Community Health Equity, Rush University Medical Center, Chicago, IL 60612, USA; joshua_longcoy@rush.edu (J.L.); zeynep_isgor-arsin@rush.edu (Z.I.); tricia_j_johnson@rush.edu (T.J.J.); 3 Department of Health Systems Management, Rush University Medical Center, Chicago, IL 60612, USA; 4 The Aspen Group, Chicago, IL 60607, USA; eyangeric@gmail.com

**Keywords:** latent class analysis, African American, health disparities, social determinants of health

## Abstract

Background: Health disparities between people who are African American (AA) versus their White counterparts have been well established, but disparities among AA people have not. The current study introduces a systematic method to determine subgroups within a sample of AA people based on their social determinants of health. Methods: Health screening data collected in the West Side of Chicago, an underserved predominantly AA area, in 2018 were used. Exploratory latent class analysis was used to determine subgroups of participants based on their responses to 16 variables, each pertaining to a specific social determinant of health. Results: Four unique clusters of participants were found, corresponding to those with “many unmet needs”, “basic unmet needs”, “unmet healthcare needs”, and “few unmet needs”. Conclusion: The findings support the utility of analytically determining meaningful subgroups among a sample of AA people and their social determinants of health. Understanding the differences within an underserved population may contribute to future interventions to eliminate health disparities.

## 1. Introduction

Disparities in health outcomes between African American (AA) people and their White counterparts in the United States (US), both historically and currently, have been well documented. The 1986 *Report of the Secretary’s Task Force on Black and Minority Health* first brought national attention to the problem and a call to action to eliminate health disparities [1]. Over 35 years later, the problems persist. Compared to White people, AA people are 60% more likely to be diagnosed with diabetes, 40% more likely to have high blood pressure, 30% more likely to be obese, and 50% more likely to have a stroke [2]. Further, they are 30% more likely to die from heart disease, nearly three times as likely to die from asthma-related causes, and have higher death rates (10–150% more likely) across the most common types of cancers [2]. Similar disparities are seen in infectious diseases, mental illnesses, infant health, and immunizations, among others [2].

The fundamental cause of these disparities is most often attributed to differences in social determinants of health that disproportionately impact AA communities [3]. Social determinants of health are non-medical conditions that affect health, such as education, working conditions, and neighborhood safety. Disproportionate exposure to social determinants of health among AA people is the downstream effect of systemic racism. For example, in education, public schools are funded partly by property taxes, favoring wealthier neighborhoods. In healthcare, access to care is determined by the ability to pay rather than need. These and other social risk factors result in conditions detrimental to health, such as segregation, a lack of safe and affordable housing, a lack of transportation, poor access to quality healthcare, and food insecurity, among others. Poverty underlies these social risk factors [4,5,6]. In 2019, the US poverty rate for AA people was the lowest in history at 18.8%, but this was still over 2.5 times higher than the 7.3% poverty rate for White people [7]. Compared to White people, AAs have a lower high school graduation rate (87.2% vs. 93.3%), a lower proportion earning at least a baccalaureate degree (22.6% vs. 36.9%), and a higher unemployment rate (8.8% vs. 4.3%) [2]. Regarding health and healthcare, AA adults under age 65 are more likely to have a disability (1 in 4 vs. 1 in 5), be uninsured (9.6% vs. 5.2%), and be unable to afford to see a doctor (19% vs. 12~15%) [8,9]. There is abundant evidence in the literature showing both independent and collective associations between these social determinants and various negative health outcomes in AA people compared to White people [10].

Interventions to improve health for AA people and other disadvantaged groups must address these powerful social determinants of health [11,12]. While these determinants disproportionately impact AA communities, they are not evenly distributed within AA communities. Moreover, social determinants of health are not independent of one another; rather, they tend to cluster together. An analysis of the 2011–2014 National Health and Nutrition Examination Survey (NHANES) data showed that, of the six different social determinants considered (education, income, insurance status, food security, housing, and employment), most were positively associated with each other as well as with race/ethnicity [13]. The same study also showed that about 30% of the 11,817 people analyzed had two or more social determinants of health [13]. Despite the clear need to target the social determinants of health, most public health interventions have targeted changing human behavior (e.g., diet and physical activity) at the individual level [12]. Although such interventions are valuable and many show positive effects, sustained change is not likely unless there are accompanying macro-level structural changes (e.g., the availability of healthy foods and safer neighborhoods) [11]. Moreover, interventions that only target a single social determinant of health (e.g., food insecurity) neglect the fact that these issues occur in clusters and that “fixing” one does not necessarily address others.

There is both evidence and widespread agreement that tailored, multifaceted interventions are needed to reduce health disparities. By disaggregating AA people into subgroups that capture patterns of variation in social determinants of health, it may be possible to better understand the mechanisms underlying health disparities and more effectively tailor interventions designed to address them. What is needed is a rigorous and empirical way to define subgroups of AA people based on how social determinants are clustered across individuals within the larger population. To this end, the current study aimed to define subgroups systematically and quantitatively within a predominantly AA sample based on 16 different social determinants of health measures by conducting a secondary data analysis on previously collected data. It was posited that the way in which the determinants cluster together would define meaningful subgroups.

## 2. Methods

Secondary data from the West Side Alive (WSA) church health screening was used. Details of the health screening and corresponding results are described elsewhere [14]. To briefly describe the population where the data originated, the West Side of Chicago, Illinois, is an urban residential area that is predominantly African American and has high poverty, low educational attainment, high rates of violent crime and homicide, as well as high levels of chronic disease. WSA is a group of churches from the West Side of Chicago within the Alive Faith Network (AFN), which is a partnership among pastors, church members, and health researchers with the goal of achieving health equity for AA church and community members. The WSA team conducted a health screening in seven partner churches to identify and prioritize health needs among adult church members and residents of surrounding communities between March and April of 2018. The only inclusion criterion was being at least 18 years of age. A total of 1106 people was screened, with 687 (62.1%) being WSA church members who reported attending church at least once a month and 419 (37.9%) from surrounding communities or with unknown church affiliation (non-WSA church members). One of the major findings from the study was that most participants reported at least one social determinant of health risk known to negatively affect health [14]. For example, 64.6% of participants perceived their neighborhoods to have a high crime rate, 47.6% were food insecure, and despite 89.9% having health insurance, 42.0% reported difficulty paying for medical services [14]. Additionally, the negative health outcomes and health risks reported from the study sample were considerably higher than those reported from AA people in the general US population. To list a few, the prevalence of hypertension was 75% vs. 61%, diabetes was 20% vs. 13%, and current smoking status was 37% vs. 21% [14].

A series of exploratory latent class analyses (LCA) were conducted to determine underlying subgroups within the sample. LCA is a statistical modeling approach used to classify participants into distinct unobserved constructs (called classes) based on observed measures (variables) [15]. The observed variables used for the LCA were selected from survey items that measured participants’ social determinants of health. Sixteen variables were used for the LCA, which are summarized in Table 1. All items were dichotomized so that the responses could be coded as either “yes” or “no”. LCA only requires that variables be categorical, but some categories were collapsed due to a lack of representation or for easier interpretability. Items in the original survey that were highly correlated or were expected to be very similar were combined to create one item. For example, a question about whether a participant can afford a specialist and a separate question about being able to afford follow-up care were combined into one. The directionality of the questions was kept consistent so that a response of “yes” indicated a negative response, e.g., “unemployed”? rather than “employed?”. Only modifiable social determinants were chosen for the analysis. Thus, demographic variables such as biological sex were not included in the LCA. However, a series of sensitivity analyses were conducted where these variables were included either as outcomes or covariates in the LCA to see if they significantly impacted the results.

LCA was run using the R statistical software with the poLCA package [16]. A total of 10 latent class models were constructed, each corresponding to a different number of classes (1–10), i.e., model 1 assumes only one latent class, model 2 assumes two latent classes, and so on. The poLCA package requires initial values for parameter estimates, but the choice of values is important as some initial values may lead to nonsensical results. Thus, for each model, 30 randomly chosen sets of initial values were used to ensure that all models were identified. The 10 models were compared using the following fit indexes: Bayesian information criterion (BIC), sample size adjusted BIC (SABIC), and the consistent Akaike information criterion (CAIC). The final model was chosen based on an iterative process of statistically identifying the best model under one or more of the fit indexes and then contextually interpreting the identified model. This process was repeated until a model with both high latent class homogeneity and high latent class separation was identified that simultaneously showed a high level of contextual interpretability. The degree to which models accurately defined classes was gauged using entropy. LCA models use maximum likelihood estimation to handle missing data intrinsically, but the missing rates for each item were generally low (<10% missing on 14 of the 16 items). To check for face validity, the relationship between class membership and demographic characteristics of age, gender, relationship status, and educational attainment was determined using chi-squared and Fisher’s exact tests, as appropriate.

## 3. Results

The latent class model with four classes had the lowest BIC and CAIC, indicating the best fit, whereas the model with six classes had the lowest SABIC. Thus, models with four, five, six, or seven latent classes were considered candidates for the final model. Although the model with seven classes did not have the lowest value for any of the fit indexes, its SABIC was very close to the SABIC from a 6-class model. Similarly, the model with five classes was considered for completeness within the range of 4–7 classes. All four models had entropy measures between 0.7 and 0.8. The four candidate models were compared using their respective conditional item response probabilities as well as their contextual interpretability. Conditional item response probabilities indicate the likelihood that a participant with that class membership would answer “yes” to a given question, hence making it more likely to have the corresponding social determinant of health as a risk factor. For models with high latent class homogeneity, probabilities close to 0 or 1 are desirable [17]. For models with high latent class separation, having distinct probability patterns for each class is desirable [18]. However, there is no convention or criteria for an acceptable threshold for probability values or probability patterns [19]. Thus, each probability was interpreted relative to other classes (i.e., low/high compared to other classes) as well as contextual implication (e.g., 0.1 is high for housing insecurity given that it is a fundamental need). The clustering of social determinants with high probabilities was used to determine the identity (labeling) of each class. As a result, the model with four latent classes was chosen as the final model. The item response probabilities for this model are shown in Table 2.

The class with the largest membership (47.1%) had no item response probability exceeding 0.2 except for having an unsafe neighborhood (which was the lowest of the four classes). Needing food stamp assistance, food insecurity, not having a primary care physician, and delaying medical care were relatively higher (0.117–0.195) compared to others but still below 0.2. As such, this class was interpreted and labeled as having “few unmet needs”. The class with the smallest membership (11.3%) had the highest probabilities for 14 out of the 16 items, with the remaining 2 (unemployment and needing food stamp assistance) being a close second. This class was labeled as having “many unmet needs”. The other two classes both had fewer unmet needs, but the needs were concentrated in distinct areas. One of these classes was labeled as having “unmet basic needs” because they tended to be unemployed, food insecure, receiving food stamp assistance, and having difficulty paying for utilities. The remaining class was labeled as having “unmet healthcare needs” because they tended to have difficulty affording dental care, eyeglasses, medications, and specialist or follow-up care and were more likely to have delayed medical care due to cost.

To investigate the face validity of the final model, at least at a rudimentary level, the relationship between class membership and other variables was examined. Table 3 shows the demographic comparisons between the four latent classes, which showed a statistically significant difference (*p* < 0.01) between class membership and each of the demographic variables. As expected, participants with many unmet needs tended to be single (64.2%) and male (54.8%) and were more likely than members of other classes to have lower educational attainment (19.3% with less than a high school education). Those with few unmet needs tended to be older (28% were older than 65) and have higher educational attainment (59.8% had post-secondary education).

## 4. Discussion

The overall aim of the current study was to define meaningful subgroups within a predominantly AA sample that capture systematic patterns of variation in social determinants of health. Exploratory latent class analysis (LCA) was conducted on a sample from an urban residential area that is predominantly AA and has high poverty, low educational attainment, high rates of violent crime, and high levels of chronic disease. Four distinct latent classes were discovered and interpreted based on their type and extent of unmet needs. These were identified as subgroups with “many unmet needs”, “unmet basic needs”, “unmet healthcare needs”, and “few unmet needs”. The findings support the utility of using latent classes as a way of identifying meaningful subgroups within AA people. Using the social determinants of health as the basis of the subgroups reveals systematic patterns of distribution among these fundamental causes that can helpfully inform interventions to address those causes and their health consequences.

One of the major contributions of the current study is that it introduces a systematic way in which subgroups can be defined to capture otherwise hidden patterns. In health disparities research, AA people are often treated as one single homogenous group while accounting for possible heterogeneity within the group by adjusting for sociodemographic factors such as age and sex. This conventional approach is fine if the goal is to establish disparities between AA and White people. Indeed, such disparities between AA and White people have been well studied and established over the years.

However, differences among AA people may be more subtle and nuanced in such a way that they are not captured entirely by the conventional approach. Case in point: the population from which the current study sample is derived is an underserved and marginalized community. As such, it is crucial to note that even the group with only “few needs” is likely to be more socioeconomically disadvantaged if a comparison to their White counterparts were possible. Likewise, it is not that the subgroup with “healthcare needs” does not have any basic needs; it is simply a matter of degree. The conditional probabilities for this subgroup for food insecurity and needing food stamp assistance were 0.446 and 0.242, respectively (Table 2). Although these probabilities were not as high as the subgroups with “many needs” or “basic needs”, they were much higher than the subgroups with “few needs”. Similarly, it is not that the subgroup with “basic needs” does not have healthcare needs. Thus, the differences between the subgroups are not necessarily huge or obvious, but the LCA model is sensitive enough to capture these subtle yet important differences.

Another major contribution of the current study is that defining subgroups based on people’s social determinants of health shows the clustering of social risk factors that are thought to directly cause health disparities. Knowing how these factors cluster may contribute to the development of more appropriately tailored interventions for underserved populations. It is worth stressing the point made above that everyone in this sample is underserved, and thus, a naïve interpretation that a certain group of people in this sample only have basic needs, for example, is incorrect and potentially harmful. The true value of the clustering of social determinants is that it elucidates a gradation of unmet needs and sheds light on why these determinants tend to cluster together. For example, the subgroup with “basic needs” is a bit younger, more male, and single compared to the subgroup with “healthcare needs” (Table 3). It is well documented that those who are older and female tend to utilize health care services more compared to their respective counterparts [20,21]. It may be that this subgroup does not seek care as often, but if they did, their healthcare needs may be similar to the subgroups with “many needs” or “healthcare needs”. In fact, the “basic needs” subgroup has the highest probability of not having a primary care physician (Table 2). On the contrary, the “healthcare needs” subgroup had a low probability of not having a primary care physician and being uninsured. Thus, it may be that they are utilizing healthcare services more but have trouble paying for them. Knowing that there are different sets of clusters of unmet needs allows for further investigation into the mechanisms causing these differences. Knowing these mechanisms will be key to the further advancement of health disparities research toward the elimination of health inequity.

A major strength of this study is that it uses locally collected data from Chicago, one of the largest cities in the US. The latent classes that were discovered through the LCA only make sense at the local level because most social determinants of health are a direct byproduct of the environment in which people live. The local context adds to the interpretability and usability of the results. For example, transportation insecurity in Chicago, where a good infrastructure for public transit is available, would be vastly different from, say, Dallas/Fort Worth, where it is not. However, that is not to say that the results are not generalizable. As of the 2020 US Census, there were about 41.1 million people who identified as AA or Black and an additional 5.8 million people who identified as a combination of two or more races, including AA or Black, in the US [22]. Of these, about 15.2 million (32.4%) live in the top 10 metropolitan areas with the highest AA population [23]. The Chicago metropolitan area is third on this list, behind New York and Atlanta. Although the specific trends may differ somewhat by area, we would expect that the way the social determinants of health cluster together should be similar across large metropolitan areas. The identified subgroups further describe the local population and the heterogeneity among AA people that cannot be captured simply by accounting for their geographic location. Another strength is the use of the LCA to systematically identify subgroups. LCA is a well-established modeling approach used in many fields and applications [24]. It qualitatively differentiates subgroups within populations with commonly observed attributes [15]. As such, it is well-suited for this application, and thus, the method can generally be applied to any underserved, marginalized population [25]. Another strength is that the subgroups that were identified can be used in future studies. The subgroups can be used as predictors in models in addition to or in lieu of race as modifiable categories of need to better capture the subtle differences within race. Alternatively, the subgroups can be used as outcomes. A longitudinal extension of LCA is called a latent transition analysis (LTA), where class membership may change over time. By using LTA, class membership itself may be used as an outcome to evaluate the progress toward health equity.

Despite the contributions and the strengths, this study is not without its limitations. One, it would have been desirable to have more granularity in the data about people’s social determinants of health risk. For example, every question about being able to afford care was asked as, “During the past 12 months, was there a time when you needed [something] but did not get it because you could not afford it?” where the possible responses were “yes” or “no”. Thus, there is no way to distinguish between people who answered “no” because they were able to afford it and those who simply did not need it. However, a series of sensitivity analyses where some or all of these questions were combined did not improve the model. Another limitation is that the WSA church screening purposefully did not ask participants about their income. As such, income was not included in any part of the analysis. It is well established that poverty is one of the strongest risk factors for premature death and other negative health outcomes [26]. However, poverty is also a strong risk factor for many of the social determinants of health. Moreover, as social determinants are the direct cause of health disparities, using them as the basis for subgrouping may be more meaningful for capturing socioeconomic position. Lastly, there was no covariate adjustment in the LCA models. Although the accuracy of class membership for an LCA model generally improves with more variables [27], the series of sensitivity analyses conducted, where variables were added as covariates to the LCA models, did not dramatically change the results.

In conclusion, the disparities between AA people and their White counterparts have been well established, but disparities among AA people have not. The current study introduces a systematic method to determine subgroups within a sample of AA people based on their social determinants of health risk. Understanding the differences within an underserved population may boost progress toward health equity.

## Figures and Tables

**Table 1:** Variables used for the latent class analysis.

Social Determinants of Health	Question
Unemployed	Is the participant unemployed?
With a disability	Is the participant disabled?
Unsafe neighborhood	Does the participant feel unsafe walking in their neighborhood during the day or night?
Food insecurity	Does the participant have trouble buying enough food?
Food stamp assistance	Is the participant currently receiving food stamp assistance?
Housing insecurity	Has the participant had difficulty finding a place to stay/live, or will they have difficulty in the next 2 months?
Utility insecurity	In the last 2 months, has the participant had difficulty paying their electric, gas, or water bill?
No health insurance	Is the participant uninsured?
No primary care physician	Does the participant lack a primary care physician?
Could not afford dental care	Has the participant had trouble affording dental care in the past 12 months?
Could not afford eyeglasses	Has the participant had trouble affording eyeglasses in the past 12 months?
Could not afford specialist or follow-up	Has the participant had trouble affording a specialist or follow-up care in the past 12 months?
Could not afford mental health care	Has the participant had trouble affording mental health care in the past 12 months?
Could not afford medications	Has the participant had trouble affording prescription medication in the past 12 months?
Delayed medical care	Has the participant delayed getting care in the last 12 months due to cost?
Transportation insecurity	Has the participant had a hard time finding transportation to and from their medical appointments?

**Table 2:** Conditional item response probabilities for answering “yes” in a four-class LCA model.

	Many Unmet Needs	Unmet Basic Needs	Unmet Healthcare Needs	Few Unmet Needs
Percentage of class membership	11.3%	25.9%	15.7%	47.1%
Social determinant of health				
Unemployed	0.296	0.298	0.015	0.040
With a disability	0.197	0.168	0.062	0.041
Unsafe neighborhood	0.818	0.808	0.656	0.592
Food insecurity	0.957	0.852	0.446	0.123
Food stamp assistance	0.704	0.751	0.242	0.195
Housing insecurity	0.280	0.187	0.000	0.032
Utility insecurity	0.678	0.506	0.302	0.069
No health insurance	0.208	0.129	0.148	0.036
No primary care physician	0.220	0.249	0.095	0.119
Could not afford dental care	0.847	0.122	0.665	0.047
Could not afford eyeglasses	0.772	0.109	0.584	0.028
Could not afford specialist or follow-up	0.829	0.067	0.424	0.011
Could not afford mental health care	0.462	0.033	0.118	0.005
Could not afford medications	0.802	0.157	0.407	0.016
Delayed medical care	0.744	0.347	0.455	0.117
Transportation insecurity	0.564	0.189	0.020	0.012

**Table 3:** Demographic characteristics overall and by subgroup.

Demographic, N (%)	Overall	Many Unmet Needs	Unmet Basic Needs	Unmet Healthcare Needs	Few Unmet Needs	*p* **
Age (n = 1099 *)						<0.01
18–29	130 (11.8)	11 (8.8)	25 (14.4)	37 (13.0)	57 (11.1)	
30–44	170 (15.5)	18 (14.4)	25 (14.4)	45 (15.7)	82 (16.0)	
45–64	565 (51.4)	85 (68.0)	77 (44.2)	172 (60.1)	231 (44.9)	
65–80	202 (18.4)	11 (8.8)	44 (25.3)	27 (9.4)	120 (23.3)	
>80	32 (2.9)	0 (0)	3 (1.7)	5 (1.8)	24 (4.7)	
Gender (n = 1091 *)						<0.01
Female	626 (57.4)	56 (45.2)	122 (70.1)	145 (51.1)	303 (59.5)	
Male	465 (42.6)	68 (54.8)	52 (29.9)	139 (48.9)	206 (40.5)	
Relationship status (n = 1035 *)						<0.01
Single	547 (52.9)	79 (64.2)	85 (48.9)	175 (63.4)	208 (45.0)	
Married or living with a partner	330 (31.9)	21 (17.1)	58 (33.3)	73 (26.5)	178 (38.5)	
Separate, divorced, or widowed	158 (15.2)	23 (18.7)	31 (17.8)	28 (10.1)	76 (16.5)	
Education (n = 1032 *)						<0.01
<high school	138 (13.4)	24 (19.3)	14 (8.0)	57 (20.6)	43 (9.4)	
High school or GED	387 (37.5)	58 (46.8)	60 (34.5)	128 (46.4)	141 (30.8)	
Post-secondary	507 (49.1)	42 (33.9)	100 (57.5)	91 (33.0)	274 (59.8)	

* n indicates the total non-missing sample size for each variable. ** *p*-value based on a chi-squared or Fisher’s exact test.

## Data Availability

The raw data supporting the conclusions of this article will be made available by the authors on request.
